# Diphenyl Ditelluride Intoxication Triggers Histological Changes in Liver, Kidney, and Lung of Mice

**DOI:** 10.1155/2015/784612

**Published:** 2015-07-05

**Authors:** Sônia Cristina Almeida da Luz, Melissa Falster Daubermann, Gustavo Roberto Thomé, Matheus Mülling dos Santos, Angelica Ramos, Gerson Torres Salazar, João Batista Teixeira da Rocha, Nilda Vargas Barbosa

**Affiliations:** ^1^Departamento de Patologia, Universidade Federal de Santa Maria (UFSM), Campus Universitário, Camobi, 97105-900 Santa Maria, RS, Brazil; ^2^Serviço de Patologia, Hospital Universitário de Santa Maria (UFSM), Campus Universitário, Camobi, 97105-900 Santa Maria, RS, Brazil; ^3^Departamento de Bioquímica e Biologia Molecular, Universidade Federal de Santa Maria (UFSM), Campus Universitário, Camobi, 97105-900 Santa Maria, RS, Brazil

## Abstract

Tellurium compounds may be cytotoxic to different cells types. Thus, this work evaluated the effect of diphenyl ditelluride ((PhTe)_2_), an organotellurium commonly used in organic synthesis, on the morphology of liver, kidney, and lung. Adult mice were acutely (a subcutaneous single dose: 250 *μ*mol/kg) or subchronically (one daily subcutaneous dose: 10 or 50 *μ*mol/kg for 7 and 14 days) exposed to (PhTe)_2_. Afterwards, the histological analyses of liver, kidney, and lungs were performed. Liver histology revealed that the hepatocytes of mice subchronically exposed to (PhTe)_2_ presented cytoplasmic vacuolization, hydropic degeneration, and hyperchromatic nuclei. Subchronic exposure to 50 *μ*mol/kg (PhTe)_2_ also caused hepatic necrosis. Microvesicular and macrovesicular steatosis were identified in liver of mice acutely exposed to (PhTe)_2_. Acute and subchronic intoxication with (PhTe)_2_ induced changes on epithelial cells of renal tubules, namely, loss of brush border and cytoplasmatic vacuolization. Atrophy and hypertrophy, cast proteinaceous formation, and acute tubular necrosis were also identified in renal tissue. Mice subchronically exposed to 50 *μ*mol/kg (PhTe)_2_ developed intra-alveolar edema and alveolar wall congestion in some areas of lungs. Acute exposure to (PhTe)_2_ did not cause histological changes in lungs. Our data show that (PhTe)_2_ may be considered a histotoxic agent for liver, kidney, and lung.

## 1. Introduction

Tellurium (Te) is a rare metalloid, which has been regarded as a toxic and nonessential trace element. It can be found in the environment as elemental and ionic inorganic forms [1, 2]. Methylation of Te inorganic forms can produce and release volatile organic forms of tellurium in the environment. Industrially, Te is obtained as by-product of copper refinement [2, 3]. Te has important applications in several industrial processes, and currently many inorganic Te compounds are used in rubber production, in metallurgy, and in the industry of nanoparticulate semiconductors [4–6]. Concerning organic Te compounds, it is important to highlight their role as reagent in organic synthesis [7]. Although Te has been known to be present in plants and microorganisms as bacteria, and fungi, there is no evidence that Te has biological functions [8]. The investigations regarding the toxicology/pharmacology of Te are still limited in literature; however, the therapeutic and toxic role of Te compounds has received more attention in the last decades. With emphasis on toxicological properties, experimental studies have highlighted the detrimental effects of different Te compounds in several tissues including liver, kidney, and blood [9–11]. Te compounds can induce severe neurodegeneration, which is strongly associated with the demyelination processes via inhibition of enzyme squalene epoxidase [1, 12, 13]. With regard to mechanisms, it has been postulated that the toxic action of Te forms (organic and inorganic) involves their prooxidant potential towards thiol groups from biologically active molecules [10, 14–19]. In a similar way, accumulating evidence has showed that the compound diphenyl ditelluride ((PhTe)_2_), an organotellurium used commonly as intermediate in organic synthesis [9], is toxic to different tissues [20–29] and inhibits sulfhydryl containing enzymes* in vitro* and* in vivo* [9, 16, 19]. Moreover, (PhTe)_2_ exposure has been associated with teratogenic, mutagenic, and genotoxic events [28–30]. Although a growing body of biochemical evidence shows the close relationship between diphenyl ditelluride intoxication and oxidative damage, there are few experimental works characterizing the putative histological changes triggered by the compound in specific mammalian organs. Only few studies have demonstrated that the exposure to certain Te compounds may induce morphological alterations in tissues such as liver, thymus, bone marrow, heart, retina, and kidney [31–33]. Specifically about (PhTe)_2_, literature data show that rats exposed to compound develop an accentuated cerebral vacuolization [1, 20]. However, the effects of (PhTe)_2_ intoxication on other target organs are still unknown morphologically. Thus, keeping in mind the (PhTe)_2_ toxicity and the scarcity of data on its action on the morphology of targets tissues such as liver, kidney and lung, the present study aimed to assess the histology of liver, kidney and lungs of mice exposed acute and subchronically to (PhTe)_2_ in order to extend, characterize and confirm morphologically the biochemical toxicity of (PhTe)_2_.

## 2. Materials and Methods

### 2.1. Materials

Hematoxylin and eosin (H&E) and Periodic Acid-Schiff (PAS) staining were purchased from and acquired from Renylab. Diphenyl ditelluride was synthesized according to the literature method [34] (Paulmier, 1986). Analysis of ^1^H NMR and ^13^C NMR spectra showed that diphenyl ditelluride presented analytical and spectroscopic data in full agreement with their assigned structures. The chemical purity of the compounds (99.9%) was determined by CGMS.

### 2.2. Animals

Adult male Swiss albino mice (25–35 g) from our own breeding colony were used. Animals were kept on a 12 h light/dark cycle, at a room maintained at constant temperature (22 ± 2°C), with free access to food and water and housed in solid plastic-bottomed cages. The animals were used according to the guidelines of the Committee on Care and Use of Experimental Animal Resources, from the Federal University of Santa Maria, Brazil.

### 2.3. Experimental Protocol

#### 2.3.1. Treatments

**Scheme 1:**
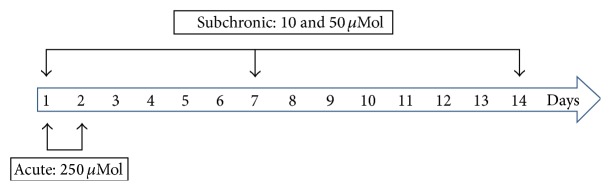


The mice were treated for different times and with doses of (PhTe)_2_ according to Scheme 1. The animals were randomly divided into control (*n* = 5) and (PhTe)_2_ (*n* = 5) groups; and the experiments were carried out 3 times. Mice in the (PhTe)_2_ groups were administered (s.c) once a day with 10 or 50 *μ*mol/kg (for 7 or 14 days) or with a single dose of 250 *μ*mol/kg of (PhTe)_2_. The compound was dissolved in DMSO and the control group was treated with the vehicle (DMSO 1 mL/Kg). The choice of (PhTe)_2_ doses used in this experimental protocol was based on a previous study [6].

#### 2.3.2. Tissue Preparation

Twenty-four hours after the end of each experimental period, the animals were euthanized by cervical dislocation. The organs designed for morphological analysis (liver, kidney, and lungs) were quickly removed, rinsed with saline solution (0.9%), and fixed in formalin 10%. The diagonal section of the liver and lung as well as the longitudinal section of the kidney was obtained and processed (Pathology laboratory, Pathology Department of Federal University of Santa Maria). The processed tissues were embedded in paraffin, sectioned at 4 *μ*m thickness, and placed on frosted glass slides for further evaluation. The tissue macroscopic alterations were also analyzed.

The samples were stained using hematoxylin and eosin (H&E) stains [35], which can detect changes in the nucleus and cytoplasm; for instance, degenerative lesions and necrosis. The slides were assessed using a light microscopy coupled to the photomicrographic camera, both adapted to a microcomputer with* software* Honestech for image capture.

## 3. Results

### 3.1. Macroscopic Analysis

**Figure 1:**
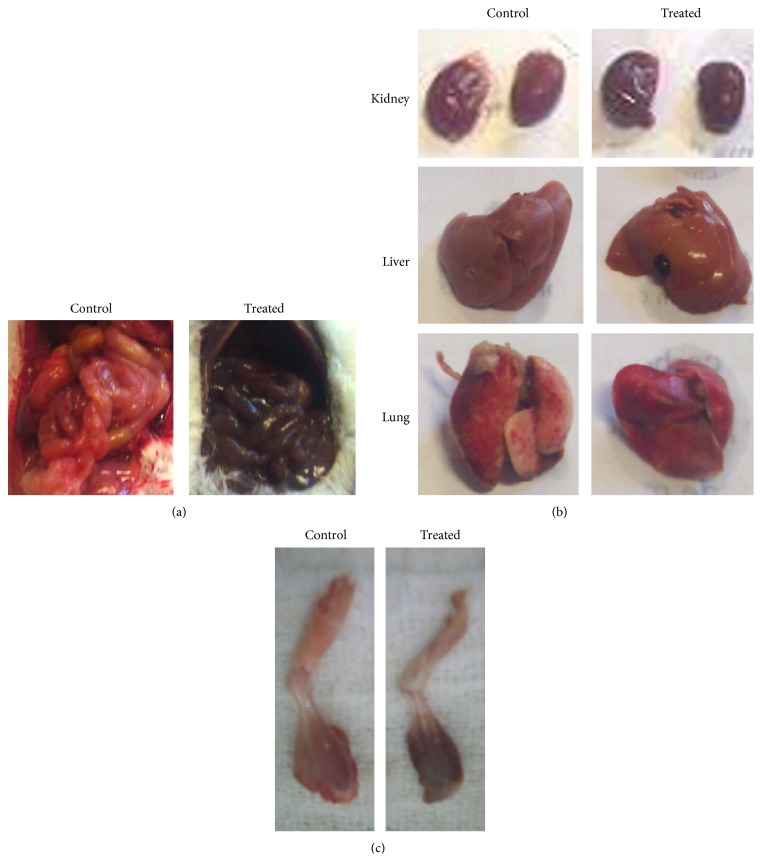
Abdominal cavity and organs of mice acutely or subchronically exposed to different diphenyl ditelluride treatments: (a) abdominal cavity of control (left) and diphenyl ditelluride (right) treated mice; (b) kidneys, liver, and lungs of control (left) and diphenyl ditelluride (right) treated mice; (c) muscles of lower limbs of control (left) and diphenyl ditelluride (right) treated mice. The picture is a representation of three independent experiments in all doses tested.

In macroscopic examination, we observed that the organs of mice exposed to (PhTe)_2_ (independently of dose and period) had a gray-black coloration. This effect was more marked in kidneys, lungs, muscles, and abdominal cavity (Figures 1(a), 1(b), and 1(c)).

### 3.2. Microscopic Analysis

#### 3.2.1. Hepatic Tissue

**Figure 2:**
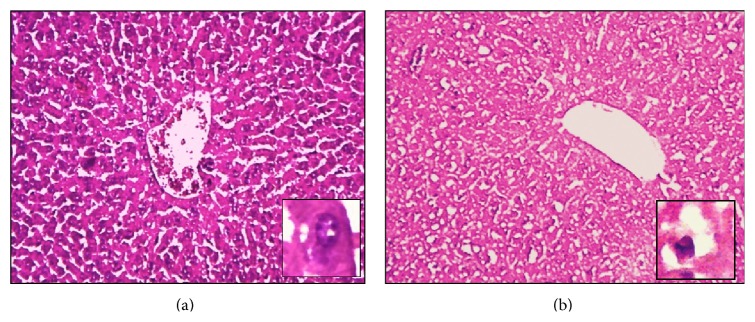
Liver histological analysis of mice exposed to diphenyl ditelluride 10 *μ*mol/kg for 7 days. (a) Hepatocytes of mice control: cells with normal nuclei, dispersed chromatin and nucleolus arranged towards hepatic central vein (detail in 40x); (b) liver section of mice treated with diphenyl ditelluride showing cytoplasmic vacuolation, edema, and hyperchromatic nuclei (detail in 40x) (H&E 10x). The picture is a representation of three independent experiments.

*(PhTe)*_*2*_ *-10 μmol/kg.* Liver histopathological analysis showed that the hepatocytes of mice exposed to (PhTe)_2_ (10 *μ*mol/kg for 7 days) presented marked cytoplasmic vacuolation (vacuoles of different sizes), hydropic degeneration (intracellular edema), and hyperchromatic nuclei when compared to the control mice (Figure 2). The same kind of morphological changes was found in the liver of mice exposed for 14 days to (PhTe)_2_ (data not shown).

**Figure 3:**
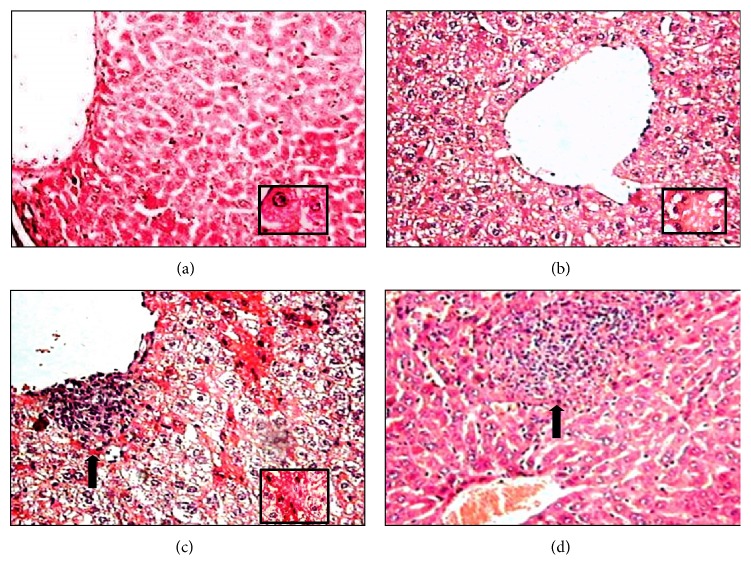
Liver histological analysis of mice exposed to diphenyl ditelluride 50 *μ*mol/kg for 7 days. (a) Liver section of control group showing polygonal hepatocytes with oval shaped nuclei, dispersed chromatin, and prominent nucleolus cordially arranged towards hepatic central vein (detail in 40x); (b) liver section of diphenyl ditelluride treated mice showing hepatocytes with manifestation of hydropic degeneration (detail in 40x); (c) pyknotic nuclei and eosinophilic cytoplasm (arrow) (detail in 40x) and (d) mononuclear infiltrate in centrilobular (zone 3) and mediolobular (zone 2) (arrow) areas (H&E 10x). The picture is a representation of three independent experiments.

*(PhTe)*_*2*_ *-50 μmol/kg.* In addition to hydropic degeneration, exposure to 50 *μ*mol/kg (PhTe)_2_ for 7 days caused hepatic necrosis (Figure 3). The signs of necrosis were evidenced by presence of hepatocytes with pyknotic nuclei and eosinophilic cytoplasm (Figure 3(c)). Aggregation of mononuclear cells in centrilobular (zone 3) and mediolobular areas (zone 2) was also found in the hepatic parenchyma (Figure 3(d)). Similar histological changes were identified in the livers of mice intoxicated with 50 *μ*mol/kg of (PhTe)_2_ for 14 days (data not shown).

**Figure 4:**
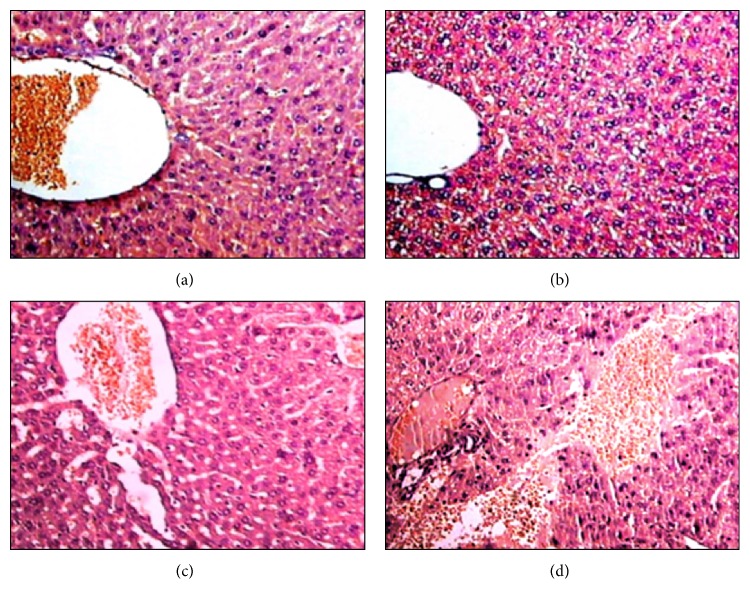
Liver histological analysis of mice acutely exposed to diphenyl ditelluride 250 *μ*mol/kg. (a) Liver section of control group showing preserved polygonal hepatocytes with oval-shaped nuclei, dispersed chromatin, and prominent nucleolus cordially arranged towards hepatic central vein; (b) liver section of diphenyl ditelluride treated mice showing microvesicular steatosis and macrovesicular steatosis; (c) presence of sinusoidal dilatation mainly in centrilobular and mediolobular areas; (d) hepatocytes with pyknotic nuclei surrounded area with vascular congestion and hepatic laminae disorganized (H&E 10x). The picture is a representation of three independent experiments.

*(PhTe)*_*2*_ *-250 μmol/kg.* Histopathologic analysis revealed that the liver of mice exposed to a single dose of (PhTe)_2_ (250 *μ*mol/kg) developed microvesicular and macrovesicular steatosis (Figure 4). Microvesicular steatosis was characterized by presence of small vesicles filling the cytoplasm of the hepatocytes (foamy hepatocytes) and nucleus localized on the cell center. Macrovesicular steatosis was characterized by large vacuoles, apparently “without filling” and rounded by a clear outline (Figure 4(b)). The acute intoxication was also associated with a marked and focal dilatation of sinusoids, which was more prominent in the centrilobular (Zone 3) and mediolobular (Zone 2) areas of the hepatic parenchyma (Figure 4(c)). In some areas a mild venous congestion and hepatocytes with pyknotic nucleus surrounding the congested area were observed (Figure 4(d)). A disorganization of hepatic laminae was also observed in this group when compared to control.

#### 3.2.2. Renal Tissue

**Figure 5:**
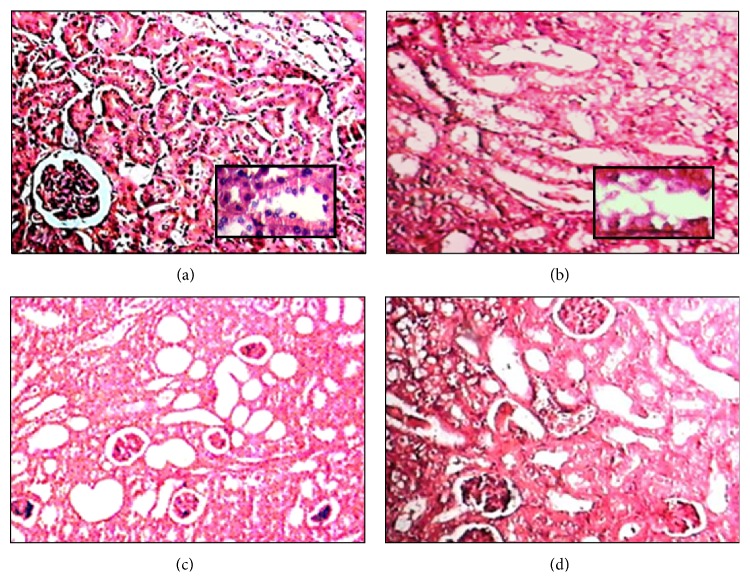
Kidney histological analysis of mice exposed to diphenyl ditelluride 10 *μ*mol/kg for 7 days. (a) Kidney section of control group showing conserved architecture of cortex with convoluted tubules outlined for a single layer of cuboidal cells and preserved glomeruli (detail 40x). Kidney section of diphenyl ditelluride treated mice showing (b) vacuolar degeneration represented for marked epithelial cells swelling of renal tubules (detail 40x); (c) dilated distal tubules and proximal tubules in different stages of compression; loss of brush border and some tubular cells free in lumen; (d) hypertrophic tubules filled with eosinophilic homogeneous substance (cast proteinaceous) (H&E 10x). The picture is a representation of three independent experiments.

*(PhTe)*_*2*_ *-10 μmol/kg.* Exposure to (PhTe)_2_ (10 *μ*mol/kg for 7 days) caused a prominent degeneration of epithelial cells lining the renal tubules (Figure 5). Degenerative processes were evidenced by presence of edema and epithelial cells with large vacuoles (Figures 5(b) and 5(c)). In several tubules signals of acute tubular necrosis were observed, for instance, an apparent loss of tubular epithelial cells specialization (brush border) and presence of necrotic debris and necrotic epithelial cells in the lumen (Figure 5(c)). Indeed, various renal tubules were filled with eosinophilic homogenous material (cast proteinaceous) and had a marked tubular hypertrophy (Figures 5(c) and 5(d)). The same kind of morphological changes were found in mice treated for 14 days (data not shown).

**Figure 6:**
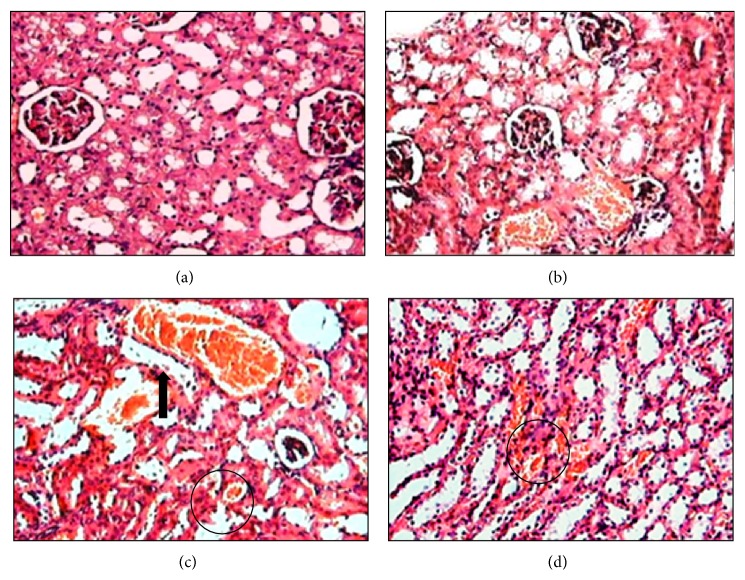
Kidney histological analysis of mice exposed to diphenyl ditelluride 50 *μ*mol/kg for 7 days. (a) Kidney section of control group showing conserved architecture of cortex with convoluted tubules outlined for a single layer of cuboidal cells and preserved glomeruli; kidney section of diphenyl ditelluride treated mice revealing (b) vacuolar degeneration represented for marked epithelial cells swelling of renal tubules; (c) hypertrophic tubules filled with eosinophilic homogeneous substance (cast proteinaceous) (arrow); and (d) presence of vascular congestion in cortical and medullar areas (circle) (H&E 10x). The picture is a representation of three independent experiments.

*(PhTe)*_*2*_ *-50 μmol/kg.* The exposure to (PhTe)_2_ (50 *μ*mol for 7 days) induced degenerative changes in the lining epithelium of renal tubules, which had cytoplasmatic vacuoles and loss of brush border (Figure 6(b)). Some tubules also presented lumen filled with eosinophilic homogenous material, characterizing the cast proteinaceous formation (Figure 6(c)). Indeed, vascular congestion in cortical and medullar areas was identified (Figure 6(d)). No additional alteration was observed in the kidney of mice exposed to (PhTe)_2_ 50 *μ*mol/kg for 14 days (data not shown).

**Figure 7:**
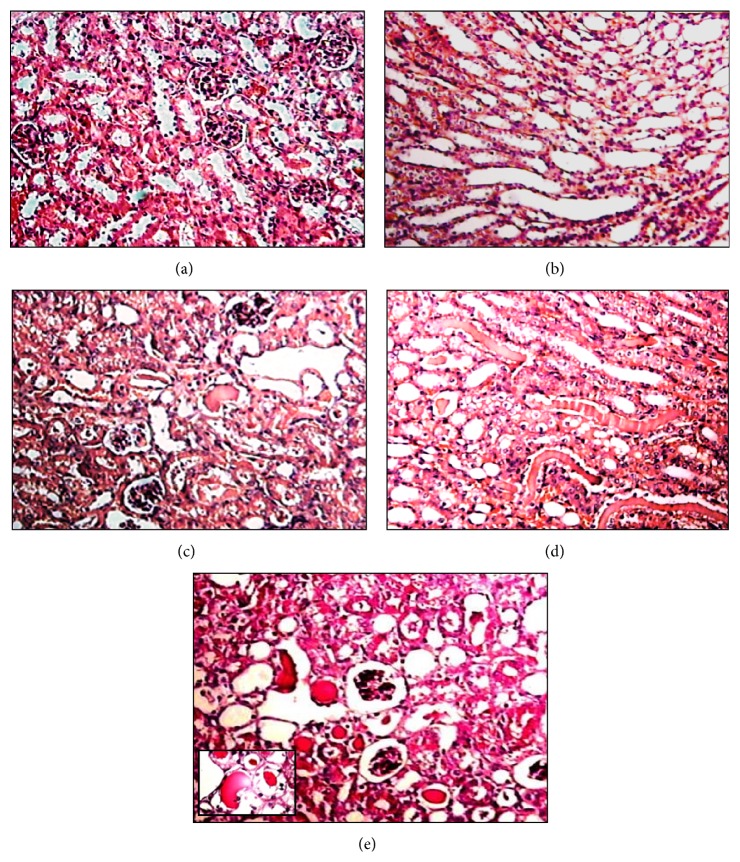
Kidney histological analysis of mice exposed acutely to diphenyl ditelluride 250 *μ*mol/kg. Kidney section of control group showing (a) conserved architecture of renal cortex with convoluted tubules outlined for a single layer of cuboidal cells and preserved glomeruli and (b) renal medullar area with collecting tubule of normal morphology. Kidney of diphenyl ditelluride treated mice showing (c) tubules in different stages of compression (arrows), epithelial cells free in tubular lumen; presence of hypertrophic tubules filled with eosinophilic homogeneous substance (cast proteinaceous); (d) presence of tubules containing a single layer of epithelial cells and small vacuoles, presence of tubules filled with eosinophilic homogeneous substance (cast proteinaceous); (e) Kidney section with PAS stain, confirming the presence of cast proteinaceous in hypertrophic tubules (positive PAS stain/detail in 40x) (H&E 10x). The picture is a representation of three independent experiments.

*(PhTe)*_*2*_ *-250 μmol/kg.* The histological analysis revealed that the renal tubules of mice exposed to a single dose of (PhTe)_2_ (250 *μ*mol/kg) contained epithelial cells in the lumen and presented different stages of compression (Figure 7(c)). Also the presence of hypertrophic tubules filled with cast proteinaceous and tubules containing a single layer of epithelial cells and small vacuoles was observed (Figures 7(c) and 7(d)). The occurrence of casts within the hypertrophic tubules was confirmed by PAS staining (Figure 7(e)).

#### 3.2.3. Pulmonary Tissue

*(PhTe)*_*2*_ *-10 μmol/kg.* No morphological alteration was observed in the pulmonary tissue of mice exposed to 10 *μ*mol/kg of (PhTe)_2_, for 7 or 14 days, when compared with the control group (data not shown).

**Figure 8:**
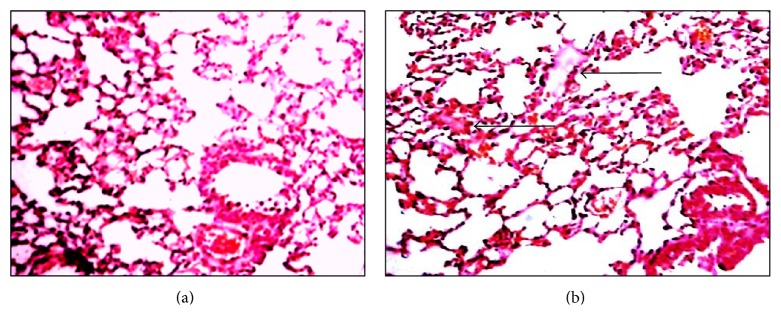
Lung histological analysis of mice exposed to diphenyl ditelluride 50 *μ*mol/kg. (a) Lung section of control group showing bronchioles, blood vessels, and adjacent alveoli with normal morphology. (b) Lung section of diphenyl ditelluride treated mice showing the presence of some isolated areas with intra-alveolar edema (arrows) (H&E 10x). The picture represents the sum of three independent experiments.

*(PhTe)*_*2*_ *-50 μmol/kg.* Lung histopathology revealed that the exposure to 50 *μ*mol/kg of (PhTe)_2_ for 7 days was accomplished by development of edema intra-alveolar and alveolar wall congestion in some areas (Figure 8(b)). Similar tissue changes were observed in the lung of mice exposed to (PhTe)_2_ 50 *μ*mol/kg for 14 days (data not shown).

*(PhTe)*_*2*_ *-250 μmol/kg.* The pulmonary analysis showed that the acute exposure to (PhTe)_2_ at 250 *μ*mol did not cause changes in alveolar morphology when compared to the control (data not shown).

## 4. Discussion

Our present work provided evidence that (PhTe)_2_ can elicit several histological abnormalities in liver, kidney, and lung of adult mice. In general, (PhTe)_2_ exposure caused degenerative lesions of reversible and irreversible character, principally in liver and kidney. In contrast, lung was little affected by (PhTe)_2_ intoxication. The macroscopic findings showed that the kidneys, lungs, muscles, and abdominal cavity of mice intoxicated with (PhTe)_2_ developed a gray-black coloration. In analogy, a blackened appearance has already been observed in the mucosa of the bladder and ureter during the necropsies analysis of a human fatally poisoned with sodium telluride [36]. In this case report, it was also emphasized that the individual intoxicated by sodium telluride presented a peculiar garlic odor in the breath and severe cyanosis [36]. Although the tissue metabolism of (PhTe)_2_ is not well studied, other studies have suggested that the black color of some tissues observed in Te intoxication fatalities possibly reflects the deposition of reduced tellurium or elemental tellurium forms [4, 36].

Currently, the toxicological properties of (PhTe)_2_ have been investigated in* in vivo* and* in vitro* experimental models. Especially in liver, acute and/or chronic intoxication have been reported to increase the organ-to-body weight ratio, inhibit *δ*-ALA-D enzyme, increase thiobarbituric acid reactive substances, decrease nonprotein SH levels, and modify antioxidant enzymes activities in rodents [17, 19, 29, 37]. In morphological terms, herein we observed that the liver of animals exposed to (PhTe)_2_ (10 and 50 *μ*mol/kg), for few days, contained hepatocytes with extensive cytoplasmic vacuolization, hydropic degeneration (edema), and hyperchromatic nuclei. The excessive accumulation of water associated with cytoplasmatic vacuolization and hydropic degeneration usually results from increased permeability of cell membranes [38]. Indeed, exposure to 50 *μ*mol/kg of (PhTe)_2_ induced a focal or nonspecific hepatitis and a focus of necrosis with dispersed cells followed by lymphocytic infiltration. Acute hepatitis with or without cholestasis is the most common histological pattern of drug-induced liver injury (DILI). It is widely recognized that DILI can be mediated by two main mechanisms: intrinsic and idiosyncratic hepatotoxicity. Commonly, intrinsic DILI is accompanied by hepatocellular necrosis and little inflammation, while the idiosyncratic DILI often with inflammation-dominant hepatic injury [39]. The liver of mice exposed acutely to (PhTe)_2_ developed marked steatosis and changes consistent with cellular necrosis such as nuclear pyknosis and dense eosinophilic bodies unaccompanied by inflammation. Acute hepatocellular injury may result in necrosis affecting a single (spotty necrosis) or groups of hepatocytes (confluent necrosis). The necrosis signals associated with (PhTe)_2_ intoxication were characterized by a confluent necrosis in centrilobular zone (zone 3), that is commonly caused by other drugs such as acetaminophen, halothane, and/or toxins like carbon tetrachloride. Frequently necrosis is accompanied by steatosis, which is characterized by presence of small fatty vesicles filling the cytoplasm of the hepatocyte (foamy hepatocyte) [40]. Here, the macrovesicular steatosis was represented by presence of single, large fat droplets in hepatocytes pushing the nucleus to the periphery of the cell. This change may be derived from the impaired egress of lipid from hepatocyte. Taken together, these sets of results indicate that (PhTe)_2_ is a xenobiotic that induces acute hepatitis and cellular death. Although there are little data on the liver histology in models of intoxication by Te compounds, our findings are in accordance with some studies that identified vacuolization and necrosis signals in hepatocytes of rats exposed to tellurium dioxide [41].

*In vivo* data on the renal deleterious action of (PhTe)_2_ are scarce in the literature. Unlike liver and brain, some biochemical analysis show that acute and/or chronic exposure to (PhTe)_2_ did not affect the activity of renal sulfhydryl enzyme *δ*-ALA-D, a marker of oxidative damage [17]. Herein, the intoxication with (PhTe)_2_ provoked several renal damage including vacuolar degeneration, atrophy and hypertrophy of renal tubules, hyaline cast formation, and acute tubular necrosis. These events reflect the cytotoxic effect of compound on renal parenchyma, which could impair the process of glomerular filtration and tubular reabsorption. Usually the hydropic changes and vacuolar degeneration appear whenever the cells are incapable of maintaining the ionic and fluid homeostasis. These features are considered the first manifestations of almost all forms of cell injury and characterize a reversible injury type [42]. In renal analysis, the atrophic aspect of tubules was distinguished by a decrease of their size following wrinkling and thickening of basal membrane. Some of atrophic tubules were also filled by cast proteinaceous, a pink mass in the lumen that corresponds to proteins filtered in glomerulus. In addition, the renal tubules of mice exposed subchronically to (PhTe)_2_ presented signals of acute tubular necrosis that was identified by presence of cytoplasm fragment projections towards tubular lumen and loosening of some of these microvesicles (“blebbing”), loss of the brush border and some free cells in the lumen. Based on these observations, it is plausible to suppose that (PhTe)_2_ exposure induced injuries on the basal membrane, the principal filtration structure, making the glomerulus abnormally permeable. In this way, there is evidence that inorganic Te compounds cause histological changes in the kidneys, ranging from cellular swelling to necrosis [31, 41]. For example, rats intoxicated with tellurium dioxide developed vacuolization of tubular cells and glomerular hemorrhage, followed by albuminuria and hematuria [31].

Regarding (PhTe)_2_ exposure and its impact in humans, it is important to consider the growing use of this organochalcogen in the workplace and consequently the increased human exposure risk [9, 34]. In this context, the knowledge about the toxicological role of compound in the lungs is extremely important, since the inhalation is the major route of intoxication in the workplace. Although this route of exposure has not been used in this work and this fact may limit our findings, the results showed here are the first pathological data reporting the effects of (PhTe)_2_ on the histology of lungs. Of toxicological importance, a recent study showed that acute exposure to (PhTe)_2_ (via s.c, 0.3, 0.6, and 0.9 *μ*mol/kg) caused oxidative damage in rat lungs, which was associated with increase in the levels of lipid peroxidation, reactive species, and nonprotein thiol as well as alterations in antioxidant enzymes activities [43]. In the histology analysis, we verified that, differing from the liver and kidney, the lung of mice intoxicated with (PhTe)_2_ by s.c route did not present signals of severe lesions. However, it was possible to observe that (PhTe)_2_ exposure induced edema and pulmonary congestion on some areas in the dose of 50 *μ*mol/kg. It has been reported that other Te forms such as cadmium telluride (via intratracheal) and tellurium hexafluoride (via inhalation) cause significant lung changes, including parenchymal inflammation, lung fibrosis, necrosis of bronchiolar epithelium, inflammation of alveolar epithelium, and lung edema [44, 45]. The differences can be explained by the route and type of compounds administered.

In conclusion, our results indicate that (PhTe)_2_ exposure provokes important morphological changes in liver, kidney, and lungs and, consequently, it represents a potential risk to human health in the work place. Although the mechanisms involved in (PhTe)_2_ responses are still under debate, our data certainly will contribute to extending the knowledge on the toxicology of (PhTe)_2_, since it is the first work that evaluates the histology of important organs after intoxication with the compound.
